# Some Remarks on the Destruction of Shade Trees as a Sanitary Measure

**Published:** 1882-02

**Authors:** Rufus W. Griswold

**Affiliations:** Rocky Hill, Conn.


ARTICLE II.

### SOME REMARKS ON THE DESTRUCTION OF SHADE TREES AS A SANITARY MEASURE.

BY RUFUS W. GRISWOLD, M. D., of Rocky Hill, Conn.

(*Concluded from January Number.*)

It is an old maxim that figures never lie. Whether this is true or not altogether depends upon how the figures are used. It is quite possible to so put them that while they seem to speak the truth they shall lie abominably. The figures I have been giving as to houses in which malarial troubles have existed, and as to other houses in which they have not, while literally true, are deceptive; now, as deception is the essential essence of falsehood, my figures lie. A very intelligent physician, driving up to my door once on a time, said: "You have got too many shade trees; I don't like them; they are unhealthy; don't you have ague?"

"Yes, badly."

"I should expect it; you ought to have these trees cut down."

"Ah! do you see that house yonder, completely shut in by trees?"

"Yes."

"They don't have ague there at all! do you see that house on the corner? And the one over there? And the one next? And the two still further below?"

"Yes."

"You see there is not a tree near one of those dwellings; into them all the sun pours and bores all day; there is not the least interruption to it."

"Yes, I see that."

"Well, in every one of those houses they have ague and fever worse than I do in mine."

"I should not have thought it."

"Not on your theory. But your theory is n't good for any thing; it doesn't hold; you may sometimes think it proven; but if you will go to the bottom of it you will discover that it is n't worth a rush Given facts upon which you build up your deductions to-day, are contradicted by another equally good set of facts that present themselves to-morrow; and your conclusion as to the causes of ague vanish. It is just as reasonable to say that my neighbor has intermittent in his family because he has n't any shade, as to say that I have it in mine because of the shade. As the actual fact, neither the trees nor the lack of them has the least connection with the disease in a single house in the town."

And this comes down to the marrow of the matter; for the truth is, that commencing with 1872, more or less of the people in three-fourths of the dwellings in the village have had ague. And they have it without any reference to their environment. On the hills as in the valley; by the bank of the river and remote from it, along the mill pond and where there is none; in the dirtiest houses and in the cleanest; in the shadow of the trees or where there is no shadow; the careless and careful alike; the sucking infant of three weeks and the tottering octogenarian, are companioned by intermittent and the cognate diseases. Even dogs have their shakes. And this in a neighborhood where what are called malarial diseases, of indigenous origin, had for generations been unknown; where it was not supposed possible for them to arise; and where the physical features of the country remain unchanged.

But, (and here comes in the error of the logic) attention being called to the existence of a case of zymotic disease in some dwelling, say of diphtheria, or intermittent, it has become the thing to look around and endeavor to find the cause of it. The head of the house calls his doctor, not only to take care of the case but to inquire into the origin of it. Do not let it be understood that the writer considers this a bad thing to do; it is all right and proper; at the same time it is well to keep your head level on the subject, and not be led astray by a false scent. One thing is certain—under the pressure of the present epidemic delusion as to the local origin of cases of fever, dysentery, diphtheria, etc., the sanitarian or the physician, who enters upon the search for a cause, *predisposed to find one*, is almost certain to discover it, or to think so, and the latter answers all his purposes. It is safe to say there does not occur a single case of what sanitarians are pleased to call filth diseases, where the investigator cannot easily convince himself that he has discovered the cause, for the utopia of perfect cleanliness does not exist. And it is amusing to note the great variety of supposed causes to which the mischief can be attributed. In the city it is a sewer, or an untrapped drain, or defective plumbing, (and how they curse the poor devil of a plumber) or a filthy street and the like. Get into the country, where they do not know what a sewer is, and never saw a plumber or any of his work; where the streets are free of filth, and so the supposed city factors can not be seized upon, and the trouble is attributed to a cess-pool, or a mill pond, or a pig stye, or a barn yard,—and if none of these are about, then something; "it may be the unemptied slop jar in my lady's house, or the solitary dump of the contented cow around the corner of the cabin; but it is a fruit," and to the fruit is attributed the evil.

Now let us look at some incontestible facts—first on one side of the subject and then on the other. It is a fact beyond controversy, that in innumerable instances of disease, there is to be found, in immediate or not very remote antiquity, some one or more of the local faults which are charged with producing mischief. They are there; their presence is not denied, and it is the first, and seems to be a proper and legitimate conclusion, that the faults are truthfully charged. To illustrate—"When one is called to a case of diphtheria, in a dirty and ill-ventilated tenement, surrounded by filth and rubbish, it is a plausible conclusion that the etiological factor is right at hand; and it is not easy to show that there is not a relation between the case of disease and its surroundings. Or again:—If one meets with an intermittent by the side of a marsh or pond, and has been indoctrinated with the theory of vegetable decomposition as the cause of agues, the next logical step seems to be that to the marsh or pond belongs the causative force. And it is with this amount and kind of evidence that perhaps the majority of minds is satisfied; a verdict is made up on the facts presented, and the surrounding coincident conditions are condemned, without looking at adverse comparisons. But if the truth is to be reached, it becomes necessary to look at the adverse comparisons, and to put them into the account. And now come the facts upon the other side of the subject. To illustrate—and taking the same diseases—when one is called to another case of diphtheria, in an isolated house, in an unexceptional location, with all the environments in the best possible condition, in the early fall of the year, and there is nothing possible to be found anywhere about like the faults to which the first case was supposed to be due, one is constrained to entertain some doubt as to the sufficiency of the evidence upon which he had made up his former judgment. Or, again, if one sees more intermittents where there is neither marsh nor pond nor any other like thing out of which they could have been evolved, he is justified in asking if really the first case came from the source supposed, or has only been assured to have come from it. Now, this is precisely the condition of things etiologically. While it is certain that an abundance of cases of disease of various kinds present themselves in contiguity with observed faulty surroundings, or surroundings supposed to be faulty, and may seem to come out of them, it is none the less certain that another abundance of cases of the like diseases thrust themselves into our notice where no similar faulty surroundings can be found out of which they could have come. And it is further certain that in innumerable instances where those supposed faulty surroundings have been present for years in precisely the same physical conditions and operated upon by the same forces from without, the diseases have been entirely absent. These two last certainties must be accounted for before we can accept the deductions too readily deduced from the first.

A critical analysis of the facts forces the more rational conclusion that some other and unrecognized element has entered in, and that to *it* and not to the old environments, is due the inflictions we observe. Putting the same thought into other words, when a typhoid, or a diphtheria, or a dysentery, or an intermittent is found in a house moss-grown because of the shadow of the ancient elm that has guarded it, perhaps an hundred years, that house having before never shown any such cases, it is unmitigated bosh to assert that the ancient elm is responsible for the disease; the charge is an unwarrantable assumption, lacking any reasonable basis of support. And yet, it is just this sort of supposed evidence against surroundings which is iterated and reiterated in the sanitary and medical journals till the repetition becomes as nauseous as the "poor polly" of the household parrot. It is just this sort of logic that is the basis of agreement with the sanitary axe-man. We need something better. When I am told that my shades are an element of unhealthiness I want some better evidence of it than the unimportant assumption of some one who evidently knows very little about the matter. I want the *proofs*, and until the proofs are forthcoming I propose to solace myself with the shadow of my vines and spruce trees, rather than by the senseless diatribes of some scribbler who knows rather less of his subject than an old maid does of the proper way to bring up a family of children.

