# Correction

**Published:** 1882-04

**Authors:** Rufus W. Griswold


### CORRECTION.

*Editor of the* INDEPENDENT PRACTITIONER :

Please correct the following errors in my article in February number : Page 81, 7th line from top, the word "fruit," twice used, should be "*find;*" same page, 12th line, "antiquity" should be *contiguity;* page 82, 2d line, "assured" should be *assumed;* 10th line from bottom, "agreement" should be *argument;* 7th from bottom, "unimportant" should be *unsupported;* 4th line from bottom, after the word *than*, insert "be governed." Correction of these will much oblige,

Respectfully, RUFUS W. GRISWOLD.

---

### RECURRING LETHARGY.

The *Lancet* records an interesting case that has been under observation at the Hospice Général, Rouen. For twenty days a woman, thirty-seven years old, has been found in a profound sleep, which is broken every night for a few minutes, when she takes food. The sleep is accompanied by general rigidity. Her first attack was fourteen years ago, and periods of lethargy have since recurred from time to time. During the intervals she seems perfectly well. The onset is always sudden : she goes to sleep in a moment, no matter what she may be doing. Just before the onset she becomes nervous and taciturn. During her sleep the face is well colored and warm, and respiration is regular. About half-past ten o'clock each night the legs begin to tremble, slight groans are uttered, and at eleven she opens her eyes, sits up and eats what is given her, without speaking. She immediately goes to sleep again (the muscular rigidity, however, not reappearing until about four in the morning), and so remains until the next night. On two occasions she has slept for ninety-six hours without food. She does not lose flesh.

